# Covalent Organic Framework Based on Azacalix[4]arene for the Efficient Capture of Dialysis Waste Products

**DOI:** 10.1021/acsami.2c06841

**Published:** 2022-08-22

**Authors:** Tina Skorjanc, Dinesh Shetty, Felipe Gándara, Simon Pascal, Nawavi Naleem, Salma Abubakar, Liaqat Ali, Abdul Khayum Mohammed, Jesus Raya, Serdal Kirmizialtin, Olivier Siri, Ali Trabolsi

**Affiliations:** † Science Division, New York University Abu Dhabi, Saadiyat Island, 129188 Abu Dhabi, UAE; ‡ Materials Research Laboratory, University of Nova Gorica, Vipavska 11c, 5270 Ajdovscina, Slovenia; § Department of Chemistry & Center for Catalysis and Separations (CeCaS), Khalifa University of Science and Technology, 127788 Abu Dhabi, UAE; ∥ Instituto de Ciencia de Materiales de Madrid-CSIC, C. Sor Juana Inés de la Cruz 3, 28049 Madrid, Spain; ⊥ Centre Interdisciplinaire de Nanosciences de Marseille (CINaM), Aix Marseille Univ, CNRS, UMR 7325, Campus de Luminy, 13288 Marseille, France; # Membrane Biophysics and NMR, Institute of Chemistry, University of Strasbourg, CNRS, Rue Blaise Pascal 1, 67081 Strasbourg, France; ¶ NYUAD Water Research Center, New York University Abu Dhabi (NYUAD), Saadiyat Island, 129188 Abu Dhabi, UAE

**Keywords:** azacalixarene, covalent organic frameworks, dialysis, uric acid, creatinine, adsorption

## Abstract

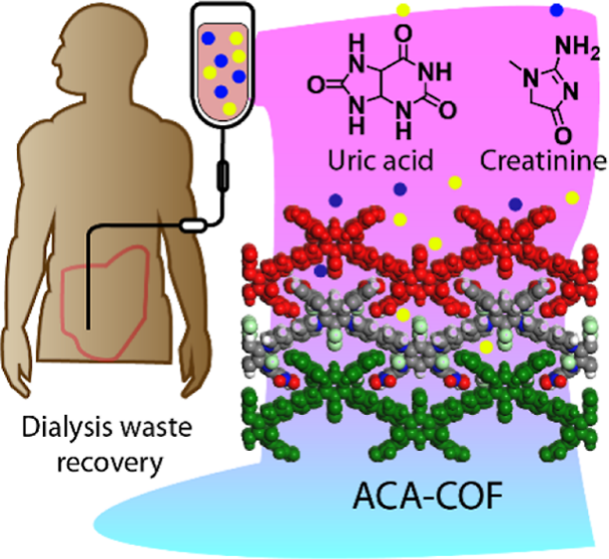

Azacalix[*n*]arenes (ACAs) are lesser-known cousins of calix[*n*]arenes that contain amine bridges instead of methylene bridges, so they generally have higher flexibility due to enlarged cavities. Herein, we report a highly substituted cationic azacalix[4]arene-based covalent organic framework (**ACA-COF**) synthesized by the Zincke reaction under microwave irradiation. The current work is a rare example of a synthetic strategy that utilizes the chemical functionalization of an organic macrocycle to constrain its conformational flexibility and, thereby, produce an ordered material. Considering the ACA cavity dimensions, and the density and diversity of the polar groups in **ACA-COF**, we used it for adsorption of uric acid and creatinine, two major waste products generated during hemodialysis treatment in patients with renal failure. This type of application, which has the potential to save ∼400 L of water per patient per week, has only been recognized in the last decade, but could effectively address the problem of water scarcity in arid areas of the world. Rapid adsorption rates (up to *k* = 2191 g mg^–1^ min^–1^) were observed in our COF, exceeding reported values by several orders of magnitude.

## Introduction

Covalent organic frameworks (COFs) are a class of purely organic, porous, and structurally ordered polymeric materials with unique predesigned two-dimensional (2D) or three-dimensional (3D) molecular architectures.^1^ The structural features of COFs depend on the rationally selected molecular building blocks, which are also reflected in the properties of the COFs. Most 2D or 3D-COFs have been demonstrated based on conformationally rigid building blocks, resulting in limited structural diversification. Recently, rigid organic macrocycles such as shape-persistent cyclodextrins^2,3^ and arylene-ethynylene^4^ have been incorporated into 3D-COFs. Although these macrocycle-associated scaffolds have diversified the library of the 3D COFs, it remains a challenge to incorporate flexible and functionalized macrocycles into COFs. The precise integration of flexible macrocycles into COFs is associated with the difficulty of maintaining order over long distances, integrating functionally diverse frameworks with chemical stability, and inducing permanent pores.^5^

With this in mind, we report here a novel macrocycle-connected COF termed **ACA-COF**. Azacalix[4]arene^6^ and the Zincke salt serve as the molecular building blocks to generate a unique 2D COF composed of highly substituted 3D building blocks. Azacalix[*n*]arenes (*n* = 3, 4, 5, 6, 8, and 10) are the structural analogues of calix[*n*]arenes with amine bridges that possess unique structural and electronic properties.^7,8^ The extended range of functional possibilities in azacalix[*n*]arenes compared to the methylene-bridged calix[*n*]arenes^9^ made them excellent candidates for the construction of macrocycle-based functional COFs. Azacalix[4]arene, a common member of the azacalix[*n*]arene family, exists primarily in the 1,3-alternate conformation in the solid state but becomes conformationally flexible in solution, where various clip-like, twisted, or ideal 1,3-alternate structures dominate depending on the phenyl ring substituents.^10^ Among azacalixarenes, only azacalix[3]arenes have been polymerized but without long-range ordering.^11^ To produce the structurally defined frameworks, we have optimized the conformational flexibility of azacalix[4]arene by introducing a specific tetranitro derivative of azacalix[4]arene monomer **1**, which is a relatively rigid version of the macrocycle that is moreover readily synthesized in a single step.^12^ In particular, the NMR studies have revealed that the 1,3-alternate conformation of **1** is locked because of the presence of N–H···O_2_N bonding interactions and the sp^2^ character of the amine bridges (strong conjugation with the nitro groups).^13^ Such an optimized lack of flexibility could play a crucial role in the highly elusive synthesis of ordered macrocycle-based open porous structures. Furthermore, **1** contains four nitro groups and four amine functionalities that can participate in various chemical reactions, including polymerizations and postsynthetic modifications.

The introduction of tetranitroazacalix[4]arene **1** into an open framework is, as yet, unknown, while its locked 1,3-alternate conformation should favor the formation of a highly organized porous framework for the specific molecular capturing. The cationic nature, macrocyclic cavities, -richness, and aromatic character of **ACA-COF** make it an excellent sorbent for highly polar aromatic molecules. Polar biological organic molecules such as creatinine (CR) and uric acid (UA) are common waste products in hemodialysis, a treatment method for patients with renal failure. An average patient undergoes hemodialysis three times a week, requiring over 100 L of water for each session, which is a huge burden in developing countries and areas suffering from drought or natural disasters.^14^ It would, therefore, be highly desirable to remove toxins from hemodialysis wastewater and reuse the purified water for various purposes such as agriculture, steam generation, or cleaning.^15^ It is estimated that treating hemodialysis wastewater with nanofiltration or reverse osmosis would be 20–30% cheaper than seawater desalination.^16^ Although hemodialysis has been used as a treatment method for over 60 years, it is only in the last decade that the conservation of wastewater produced during the treatment is being considered and researched. As a result, not many methods have been developed to solve the problem. Enzymatic and electrochemical degradation of dialysis waste products have been studied, but potentially harmful byproducts may be generated.^17^ In contrast, adsorption onto solid materials has been considered scalable, reversible, and affordable although very few sorbents have been studied specifically for dialysis wastewater treatment.^18,19^ In particular, the unique structural and chemical features of **ACA-COF** promote the efficient adsorption of these biological molecules from water through the formation of hydrogen bonds, ion-dipole, and π–π stacking interactions. Performing adsorption experiments with **ACA-COF**, we obtained excellent adsorption rates with rate constants of *k* = 1333.03 g mg^–1^ min^–1^ for UA and *k* = 2191.03 g mg^–1^ min^–1^ for CR. We studied the binding properties of these biologically relevant molecules to **ACA-COF** using MD simulations, and found that the material preferentially removed UA over CR from water due to the development of electrostatic and π–π interactions. We also identified several regions of **ACA-COF** where the binding events were most favorable.

## Results and Discussion

**Figure 1:**
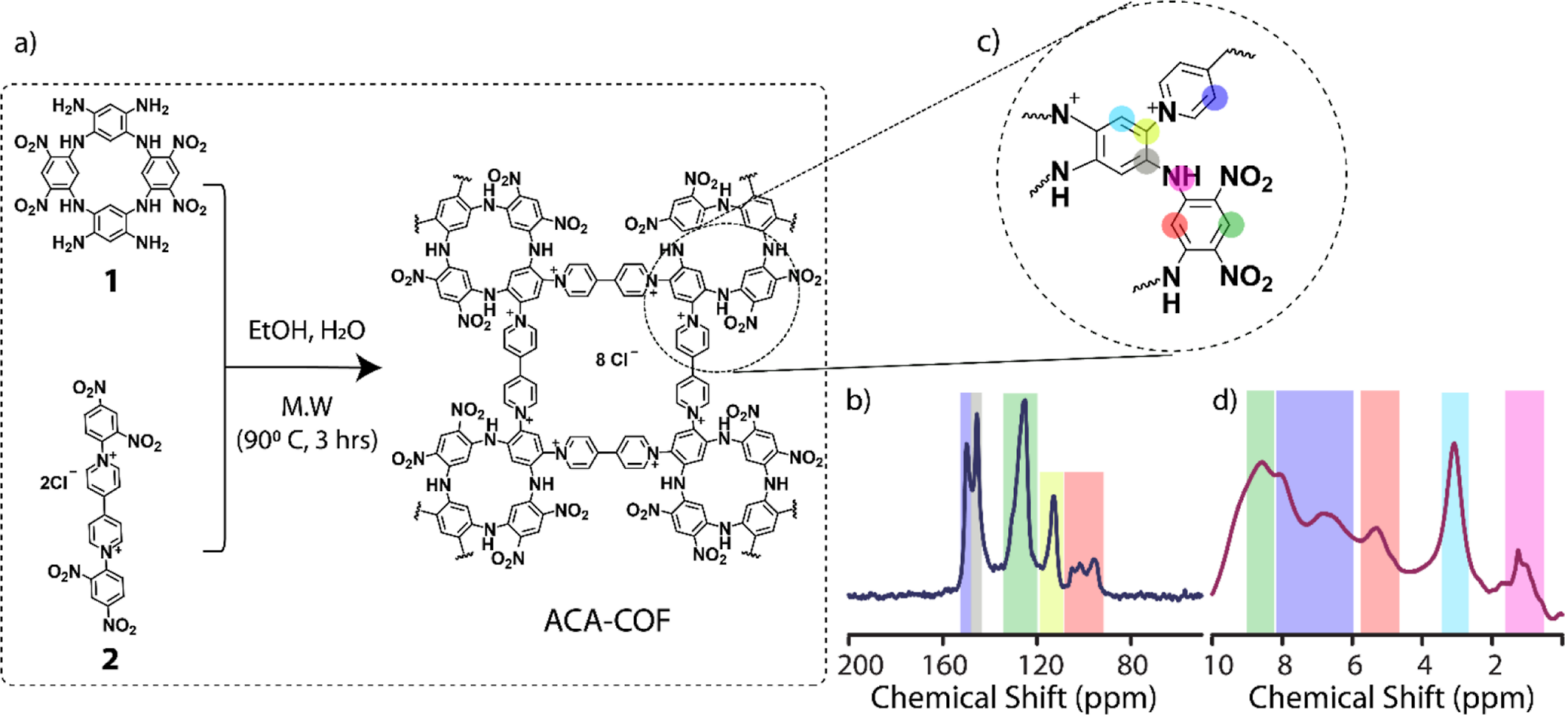
Design strategy for cationic **ACA-COF** through the Zincke reaction (a); ^13^C (b) and ^1^H (d) CP-MAS solid-state NMR spectra of **ACA-COF** with peaks assigned in panel (c).

The **ACA-COF** was synthesized from tetranitro tetraamino-substituted ACA (**1**) and bipyridinium salt (**2**) through the microwave-assisted Zincke reaction. The resulting chestnut brown powder was purified by Soxhlet extraction using water and ethanol as solvents and dried in a vacuum oven overnight (Figure 1a, details in Supporting Information). It is noteworthy to mention that the reaction shown in Figure 1a was attempted in solvothermal conditions, but the level of crystallinity observed was poorer than for the product synthesized under microwave irradiation. This latter technique has previously been noted for favoring crystallinity in the Zincke reaction over solvothermal synthesis.^20,21^

The formation of the polymeric structure was verified by Fourier-transform infrared (FT-IR) spectroscopy: The FTIR spectrum of **ACA-COF** did not exhibit the N–O signals present in the starting Zincke salt **2** at 1340 and 1530 cm^–1^. In addition, a shift of the N–H signal from 3340 cm^–1^ in the starting ACA **1** to 3330 cm^–1^ in **ACA-COF** was observed (Figure S1). At ∼1240 cm^–1^, aromatic C–N stretching occurs in **ACA-COF**, but not in the starting ACA. The chemical distribution at the atomic level of **ACA-COF** was investigated by ^1^H and ^13^C CP-MAS spectra (Figure 1b–d). The ^13^C NMR spectrum shows peaks corresponding to the two building blocks of **ACA-COF**. The aromatic C atoms of the linker appear at 150.4 ppm (purple). The C atoms of ACA facing the cavity are visible in the 95.4–105.4 ppm range (red). NO_2_ groups and pyridinium N^+^ atoms in the linker cause deshielding of C atoms, so that their signals appear at 145.6 (gray) and 125.7 ppm (green), respectively. These structure characterizations at the molecular level clearly indicate the formation of an extended structure in **ACA-COF** (Figure 1a).

The material exhibited the morphology of 2D patches or sheets, as seen in scanning electron microscopy (SEM) and transmission electron microscopy (TEM) micrographs (Figure S2). SEM images showed clumps of μm-sized particles with no distinct morphology, whereas the TEM images suggested that these particles consisted of thin sheets. Thermogravimetric analysis (TGA) was carried out by first equilibrating the samples at 70 °C to remove any trapped solvents, and then, the temperature was ramped up to 1000 °C. **ACA-COF** exhibits enhanced thermal stability up to 350 °C compared to its building blocks (Figure S3). Although the final weight loss at 1000 °C was greater for **ACA-COF** than for ACA, the thermal stability of the COF was comparatively higher up to 350 °C. Such a trend has previously been observed in other macrocycle-based COFs.^20^ The ζ-potential of **ACA-COF** in water was measured to be +39.8 mV (Figure S4). In addition, the porosity features of **ACA-COF** were analyzed by recording N_2_ adsorption isotherms at 77 K (Figure 3a). **ACA-COF** was moderately porous with a specific BET surface area of 58 m^2^ g^–1^. This relatively modest surface area could be explained by the presence of counterions known to decrease the surface area of COFs synthesized by the Zincke reaction.^20,21^ Based on the pore size distribution, the material shows micropores with an average diameter of 1.2 nm as well as mesopores with an average diameter of 7.5 nm (Figure S5). This pores-within-pores property can be attributed to the pores of the macrocycle as well as the pores of the COF and has been observed in other calixarene-based systems.^22^

**Figure 2:**
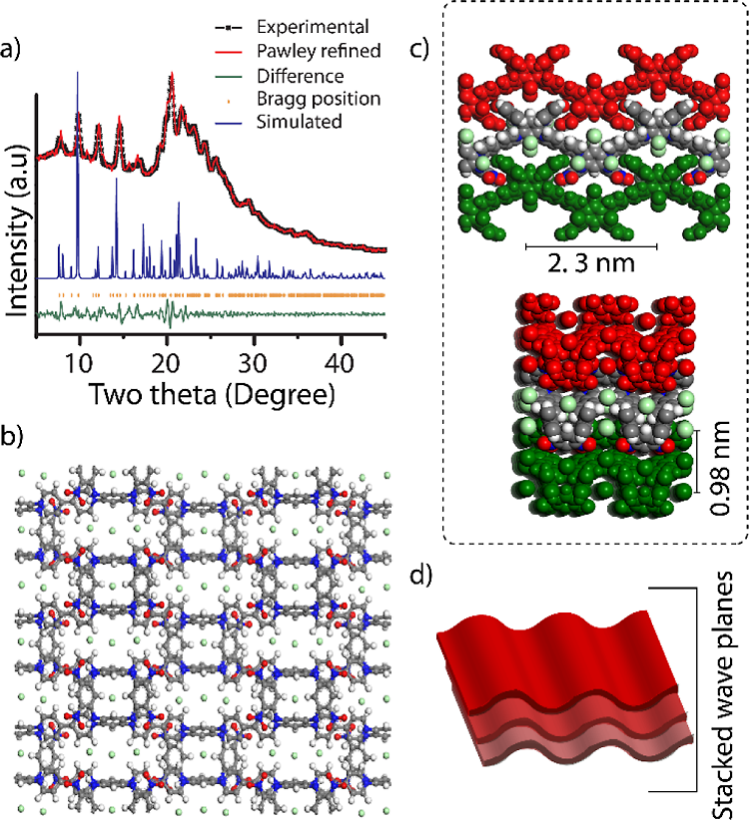
Structure of **ACA-COF**. Experimental and Pawley-refined PXRD patterns, along with the diffraction lines calculated for the optimized model (a); structural models as viewed along the **a**-axis and **b**-axis with associated unit cells parameters displayed (b,c); schematic representation of layer stacking in **ACA-COF** (d).

The powder X-ray diffraction (PXRD) profile of **ACA-COF** with sharp crystalline peaks indicates the long-range order and periodicity of the framework. The main peaks were found at 2θ = 7.9, 9.9, 12.1, 14.5, and 20.5° (Figures 2a and S6). A crystal model was created based on the geometry of the ACA building unit, where the terminal amino groups determine the position of four points of extension connected by the linear bipyridium units. This leads to the formation of square layers, which, in this case, have a wave-like conformation originating from the macrocyclic nature of the ACA molecules. The corresponding crystal model was completed in the orthorhombic *Pmmn* space group. The initial unit cell parameters were obtained from indexing the experimental PXRD pattern and, then, refined with the Pawley procedure (*a* = 23.30 Å, *b* = 12.46 Å, *c* = 9.84 Å, *R*_p_ = 1.98%, and *R*_wp_ = 2.68%). The crystal model was geometrically optimized using forcefield-based energy minimization procedures. The required number of chloride counter ions was introduced in the unit cell occupying the internal pores. The model shows that each **ACA-COF** unit is connected to four other units that have an inverted arrangement with the nitro groups pointing in opposite directions (Figure 2b,c). The layers are stacked along the [001] direction. In addition to the diffraction peaks observed both experimentally and by modeling, a broad peak between 2θ = 20° and 2θ = 25° can be observed. We attribute this peak to π–π stacking that formed between the aromatic layers (Figure 2d).^23^

Prior to utilizing **ACA-COF** in dialysis waste product removal, its stability in water and phosphate-buffered saline (PBS) was investigated. 20 mg of **ACA-COF** was suspended in 5 mL of either medium, briefly sonicated, and stirred at room temperature for 48 h. After the incubation, the sample was dried and analyzed with FT-IR, SEM, and PXRD. FT-IR spectra of the native and water- or PBS-treated samples showed highly similar bond vibrations (Figure S7). The PXRD pattern (Figure S8) and the morphology (Figure S9), likewise, remained unaffected by the 48 h exposure to either medium. Overall, these results suggested that **ACA-COF** is stable in water as well as in biological fluids, which encouraged us to proceed with adsorption experiments.

**Figure 3:**
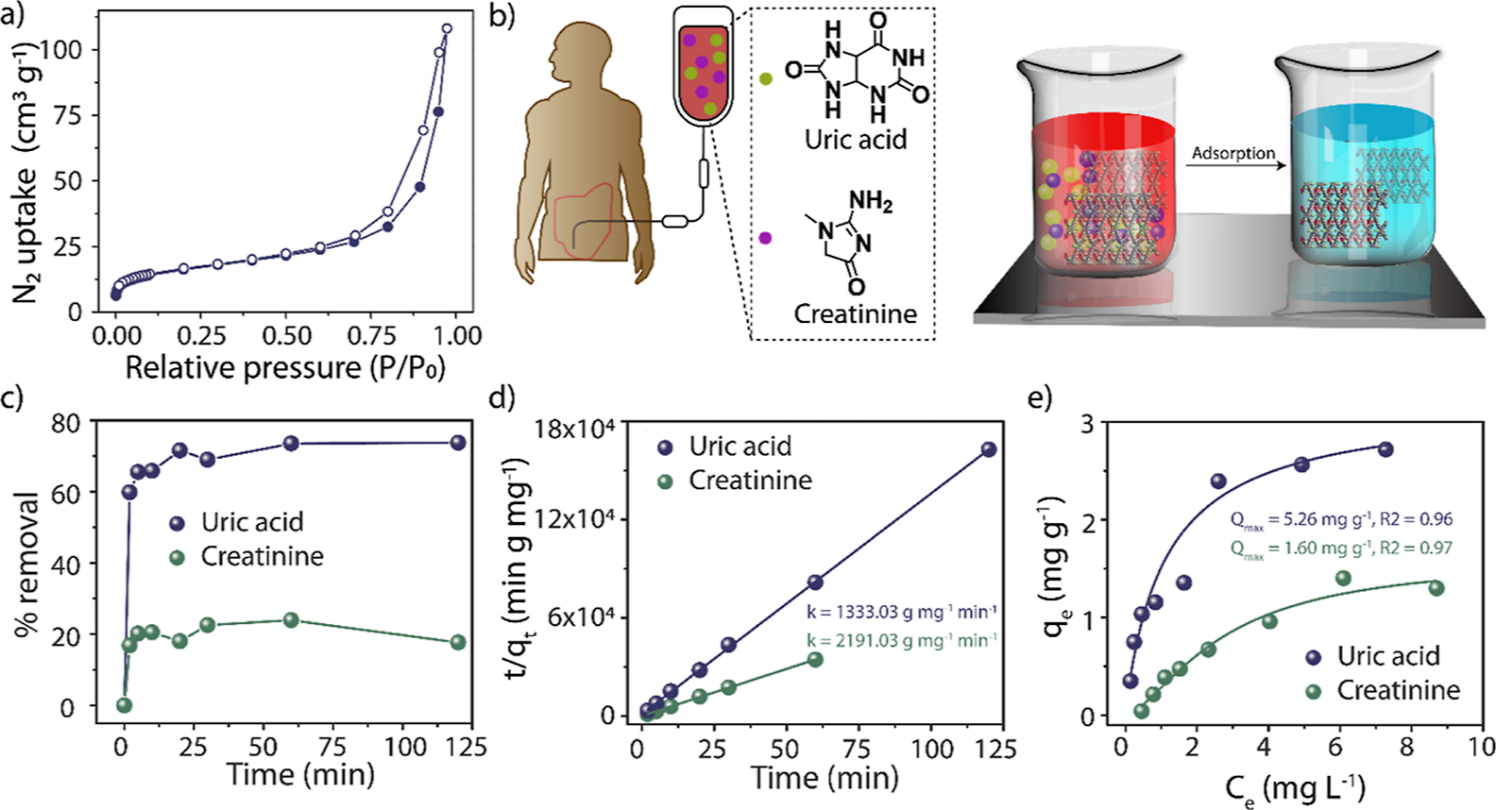
Adsorption experiments. N_2_ gas adsorption isotherm for **ACA-COF** (a); schematic representation of dialysis waste product adsorption onto **ACA-COF** (b); percent removal of uric acid (blue) and creatinine (green) by **ACA-COF** (c); pseudo-second-order fit of the kinetics data with associated rate constants displayed (d); isotherm for uric acid and creatinine adsorption by **ACA-COF** with associated *Q*_max_ values (e).

The specially designed **ACA-COF**, rich in polar functional groups, exhibits excellent adsorption properties toward highly polar UA and CR molecules (Figure 3b). These chemicals are present in substantial amounts in large volumes of wastewater produced in hospitals that dialyze patients with renal dysfunction. An average dialysis session lasts 4 h and requires a flow rate of 500 mL min^–1^, thus consuming 120 L of purified water.^16^ Patients with renal failure have blood concentrations of CR^24,25^ and UA^24^ of 8–10 mg dL^–1^, whereas in healthy individuals, these levels are 0.6–1.2 and 2.4–6 mg dL^–1^, respectively. Considering that an average adult has a blood volume of about 5 L, it can be assumed that the concentrations in dialysis wastewater are about 2.8–3.9 and 0.8–2.9 mg L^–1^, respectively. Considering these values, we performed adsorption experiments with 1.0 mg L^–1^ UA and 2.5 mg L^–1^ CR solution. **ACA-COF** removed ∼70% of UA and 24% of CR within 120 min (Figure 3c). The kinetic adsorption experiments, in which an aliquot was taken and the presence of either pollutant was quantified at different time points, were used to fit the data to the pseudo-second-order kinetic model and determine the rate constants (*k*) of adsorption shown in Figure 3d. Exceptionally high *k* values of 1333.03 g mg^–1^ min^–1^ for UA and 2191.03 g mg^–1^ min^–1^ for CR adsorption indicate rapid removal of both pollutants. This property is particularly important for materials incorporated into membranes. The high uptake rates indicate that even a brief interaction of the polluted water with the COF is sufficient for the removal of the pollutants. From the literature, it appears that the kinetic aspect of UA adsorption is rarely studied. However, **ACA-COF** removes CR at a rate that is several orders of magnitude higher than that of other reported adsorbents, which include activated carbons, polymers, hybrid materials, zeolites, and metal–organic frameworks (Tables S1 and S2). We postulate that such high rates of adsorption are a result of the pollutant concentrations used. It has been observed that lower initial CR concentrations result in higher rate constants,^26^ but most reported studies employ concentrations well above those relevant to the dialysis wastewater treatment. In addition, the maximum adsorption capacity of **ACA-COF** was calculated for UA and CR. The Langmuir isotherms indicate that the *Q*_max_ values are 5.26 and 1.60 mg g^–1^, respectively (Figure 3e).

**Figure 4:**
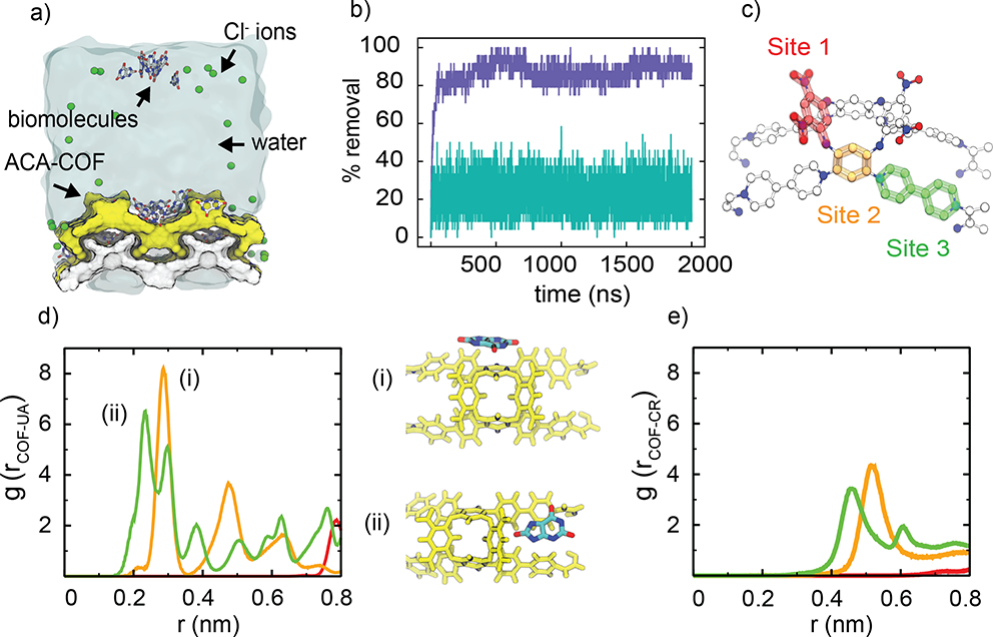
(a) MD simulation setup of **ACA-COF** with biomolecules in explicit water and ions. (b) Uptake capacity monitored by the time evolution of percent removal of biomolecules from bulk; purple line for uric acid and green line for creatinine. (c) Identified regions on **ACA-COF** for biomolecule binding. (d,e) Radial distribution functions of the biomolecules, with each region colored according to the binding sites shown in (c): (d) for uric acid and (e) for creatinine. The inset in (d) shows the representative binding of biomolecules at the peak position.

To understand the interactions between the biomolecules and **ACA-COF**, we constructed a model system consisting of the molecules of UA and CR and **ACA-COF** layers in explicit water and ions (Figure 4a). We performed molecular dynamics (MD) simulations to gain an insight into the binding mechanisms of UA and CR to **ACA-COF**. For each system, 24 ligand molecules were added to the simulation box of 50 Å × 25 Å × 60 Å (details in Supporting Information). Two microsecond MD simulations were used to capture the distributions of the small molecules. We estimate the percent removal of molecules from the simulations when each system reaches equilibrium (Figure 4b). The percent removal was estimated based on the number of the small molecules bound to **ACA-COF**. Molecules within 6 Å of the surface of **ACA-COF** were defined as bound. Consistent with experimental trends, UA was adsorbed more strongly to the COF, with an average removal capacity of 86% from an aqueous UA solution. For CR, this value was found to be ∼22%. Note that the experimental time scales in Figure 3 differ from the time scales of the simulations. This is due to the differences in concentration and the finite size of the **ACA-COF** used in the simulations.

As dialysis wastewater includes a high concentration of salt, it is important to also check the performance of the **ACA-COF** under such conditions. To investigate the salt effects, we extended the study to saline conditions in both adsorption experiments and MD simulations. Relevant to real dialysis wastewater concentrations, we employed solutions containing 150 mM NaCl and the same concentration of UA and CR as in the kinetics experiments (1 and 2.5 mg L^–1^, respectively). Despite the presence of competitive salt ions, no reduction in the removal of UA was observed (Figure S10). We also simulated the same systems at 150 mM NaCl aqueous solution. Simulation results are shown in Figure S11 in comparison to the no salt case in Figure 4b. We observe no detectible difference between saline and no salt conditions.

To obtain atomic details of how these molecules bind to **ACA-COF**, we studied their distributions using the radial distribution function (RDF). We divided the surface of the repeating unit of **ACA-COF** into three regions (Figure 4c). Site 1 is the aromatic ring in ACA containing two NO_2_ groups, site 2 is the −NH_2_ functionalized aromatic ring in ACA that reacts during the polymerization, and site 3 is the aromatic group of the bipyridinium linker. The RDF between the UA and each of these sites shows differences indicating preferential binding at the surface (Figure 4d,e). The UA molecules bind strongly to the **ACA-COF** linker region (Figure 4d inset). At this site, the electrostatic interactions between **ACA-COF** and charged UA determine the specific binding. In addition to the linker, we observe a notable interaction with site 2, where the small molecules are sandwiched between adjacent aromatic rings and form a π–π stacking interaction. Site 1, on the other hand, is the least preferred region of **ACA-COF** for binding due to its negative partial charge localized at the polar NO_2_ groups. CR binding shows similarities to UA. However, lower intensities in RDF with wider spacings indicate weaker interactions (Figure 4e).

## Conclusions

In summary, a highly substituted cationic COF, containing tetranitroazacalix[4]arene subunits, was demonstrated to adsorb biomolecules from water. The introduction of macrocycles locked in their rigid 1,3-alternate conformation into a COF promoted a wave-like structure with open porosity that enhanced the adaptive capturing of biomolecule pollutants. The high density of the nitro functional groups in **ACA-COF**, which are not involved in the polymerization reaction, gives this material potential for postsynthetic modification and provides polar sites for interaction with guest-pollutant molecules. We have utilized the bipyridinium and secondary amino functional groups, macrocyclic cavities, and the porosity in the removal of pollutants from dialysis wastewater with high adsorption kinetics (*k* up to 2191 g mg^–1^ min^–1^). The efficiency of CR and UA adsorption was investigated by modeling studies, which showed that **ACA-COF** has two regions for favorable interactions with the selected biomolecules. The aromatic ring of the linker has a net positive charge, which leads to strong charge–charge and π–π interactions with negatively charged UA. This site is less favorable for CR, as it has zero net charge. The aromatic ring on the cone provides a favorable binding site for both small molecules. At this site, UA has a larger surface area leading to stronger binding compared to CR. These experimental and theoretical observations on the high adsorption kinetics and selectivity of UA adsorption over CR should pave the way to more sustainable and efficient hemodialysis water purification systems.

## Supporting Information Available

Monomer and COF synthesis procedures, FT-IR spectra of the starting materials and COFs, SEM and TEM characterization of the COFs, TGA profiles of the monomers and COFs, ζ-potential measurements, and pore-size distributions, water and buffer stability testing and characterization, crystal structure details, details of the adsorption experiments, performance of known adsorbents, and details of the MD simulations (PDF)

## Abbreviations

ACA: azacalix[4]arene
BET: Brunauer–Emmett–Teller
COF: covalent organic framework
CR: creatinine
FT-IR: Fourier transform infrared spectroscopy
MD: molecular dynamics
NMR: nuclear magnetic resonance
PXRD: powder X-ray diffraction
SEM: scanning electron microscopy
TEM: transmission electron microscopy
UA: uric acid
