# Supplementary material for: Covalent Organic Framework Based on Azacalix[4]arene for the Efficient Capture of Dialysis Waste Products
Source: ACS Appl Mater Interfaces. 2022 Aug 22;14(34):39293–8. doi: 10.1021/acsami.2c06841 (PMC9437870; doi:10.1021/acsami.2c06841)
Supplement: Supplementary file 1 — am2c06841_si_001.pdf [file am2c06841_si_001.pdf]

Supporting Information

Covalent organic framework based on azacalix[4]arene for the efficient capture of dialysis waste products

Tina Skorjanc,[a,b,‡] Dinesh Shetty,[*c,‡] Felipe Gándara,[d] Simon Pascal,[e] Nawavi Naleem,[a] Salma Abubakar,[a] Liaqat Ali,[a] Abdul Khayum Mohammed,[c] Jesus Raya,[f] Serdal Kirmizialtin,[a] Olivier Siri,[*e] and Ali Trabolsi[*a,g]

[a] Science Division, New York University Abu Dhabi, Saadiyat Island, Abu Dhabi, UAE

[b] Materials Research Laboratory, University of Nova Gorica, Vipavska 11c, 5270 Ajdovscina, Slovenia

[c] Department of Chemistry & Center for Catalysis and Separations (CeCaS), Khalifa University of Science and Technology, P.O. Box 127788 Abu Dhabi, United Arab Emirates

[d] Instituto de Ciencia de Materiales de Madrid-CSIC, C. Sor Juana Inés de la Cruz 3, 28049 Madrid, Spain

[e] Centre Interdisciplinaire de Nanosciences de Marseille (CINaM), Aix Marseille Univ, CNRS, UMR 7325, Campus de Luminy, 13288 Marseille, France

[f] Membrane Biophysics and NMR, Institute of Chemistry, University of Strasbourg – CNRS, Rue Blaise Pascal 1, Strasbourg, France

[g] NYUAD Water Research Center, New York University Abu Dhabi (NYUAD), Saadiyat Island, P.O. Box 129188, Abu Dhabi, United Arab Emirates

‡ These authors contributed equally.

*Correspondence: dinesh.shetty@ku.ac.ae, olivier.siri@univ-amu.fr, ali.trabolsi@nyu.edu

General and materials characterization

Routine nuclear magnetic resonance (NMR) spectra were recorded at 25 °C on a Bruker Avance spectrometer, with working frequency of 500 MHz for ^{1}H and 127 MHz for ^{13}C{^{1}H}.. All chemical shifts are reported in ppm relative to the signals corresponding to the residual non-deuterated solvents (CD_3OD: δ = 3.31 ppm). The multiplicity is abbreviated in the following way: s (singlet), d (doublet), m (multiplet). FTIR studies were carried out on Agilent 670 IR spectrometer in the attenuated total reflectance (ATR) mode. TGA experiments were performed on a TA SDT Q600 with a heating rate of 10 °C min^{-1} over a temperature range of 25–1000 °C. Powder X-ray diffraction (PXRD) measurements were carried out on Bruker D8 Advance X-ray diffractometer with Cu K_α (λ = 1.5405 Å) radiation source operating at 40 kV and 30 mA. The patterns were recorded with divergent slit of 1/16° over the 2θ range of 1–50° with step size = 0.01°. Surface area measurements were conducted on a Micromeritics 3Flex gas sorption analyzer. Samples (~30 mg) were degassed at 85 °C for 20 h and then backfilled with N_2. Adsorption isotherms were generated by incremental exposure to ultrahigh-purity nitrogen up to 1 atm in a liquid nitrogen bath, and surface parameters were determined using BET adsorption models included in the instrument software (Micromeritics ASAP 2020 V4.00). SEM images were obtained from FEI Quanta 450FEG. TEM images were obtained from a FEI-Titan 300 operating at 200 kV. Dynamic light scattering (DLS) measurements were performed on a Malvern Zetasizer NanoSeries.

Synthetic procedures

Azacalixarene (**1**) was synthesized according to a previously published procedure.[1]

1,1'-bis(2,4-dinitrophenyl)-[4,4'-bipyridine]-1,1'-diium dichloride (Zincke salt, **2**) was synthesized according to a published procedure.[2] In brief, 4,4'-bipyridine (4 g, 25.60 mmol, 1 equiv.) and 1-chloro-2,4-dinitrobenzene (26 g, 89.60 mmol, 3.5 equiv.) were refluxed in 150 mL of anhydrous acetonitrile under Ar for 72 hours. After the reaction was complete, the mixture was filtered, and the solid was washed with acetonitrile (50 ml, once) and diethyl ether (40 ml, four times, followed by soaking) to afford the pure Zincke salt in 75% yield. ^{1}H NMR (400 MHz, MeOD): δ 9.46 (d, 4H), 9.39 (d, 2H), 8.90–8.94 (m, 6H), 8.29 (d, 2H) ppm. ^{13}C{^{1}H} NMR (126 MHz, D_2O) δ 152.7, 149.9, 146.9, 142.8, 138.2, 131.1, 130.8, 127.6, 122.8 ppm.

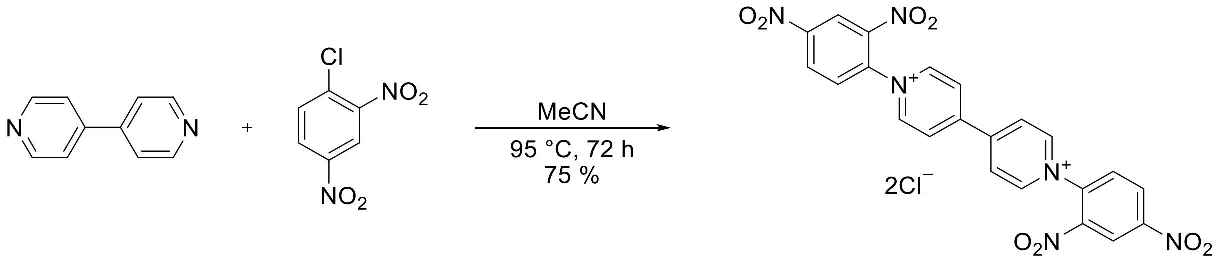

2

ACA-COF was synthesized by reacting 200 mg of **1** (0.331 mmol, 1 equiv.) with 372 mg of **2** (0.663 mmol, 2 equiv.) in a 1:1 mixture of ethanol:water (20 mL) under microwave irradiation at 90 °C for 3 hours. The product, which precipitated out of the reaction mixture, was purified by Soxhlet extraction using first water followed by ethanol, and dried in a vacuum oven at 45 °C overnight. The purified material was obtained in reasonable yield with ~300 mg obtained per batch.

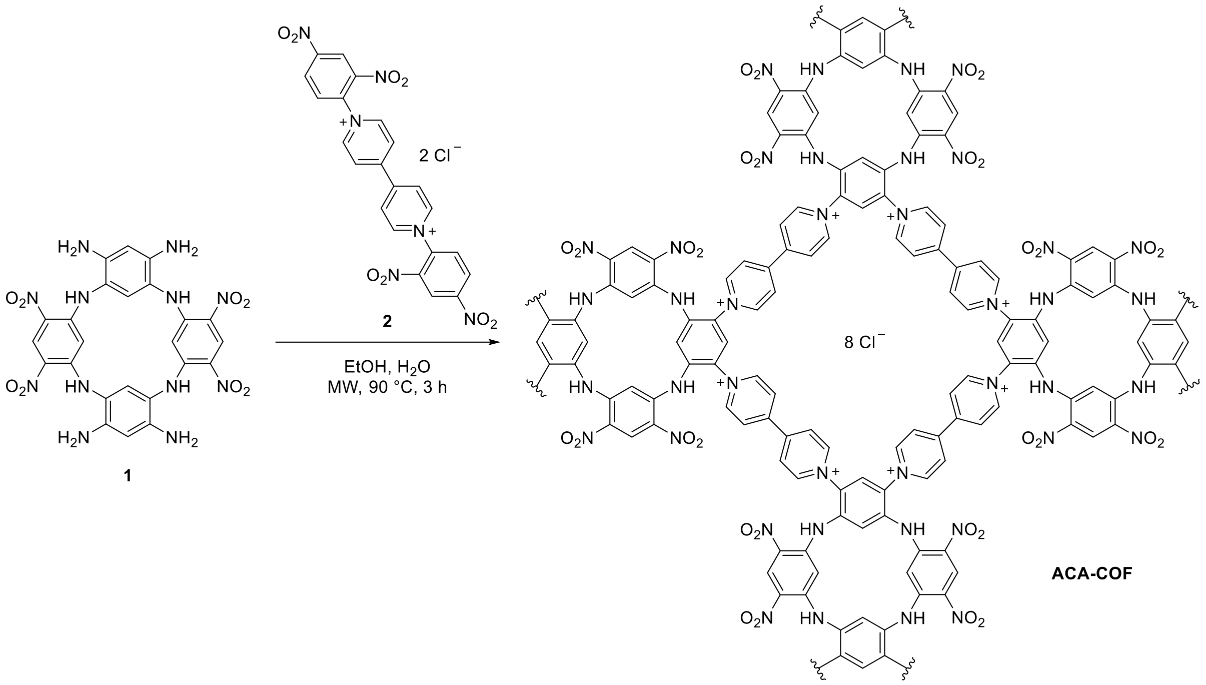

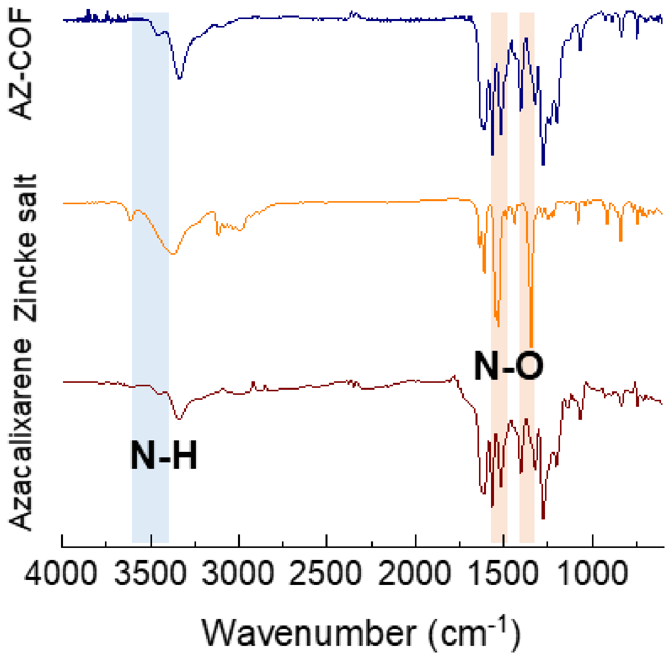

Figure S1. FTIR spectra of **ACA-COF**, and the corresponding starting materials.

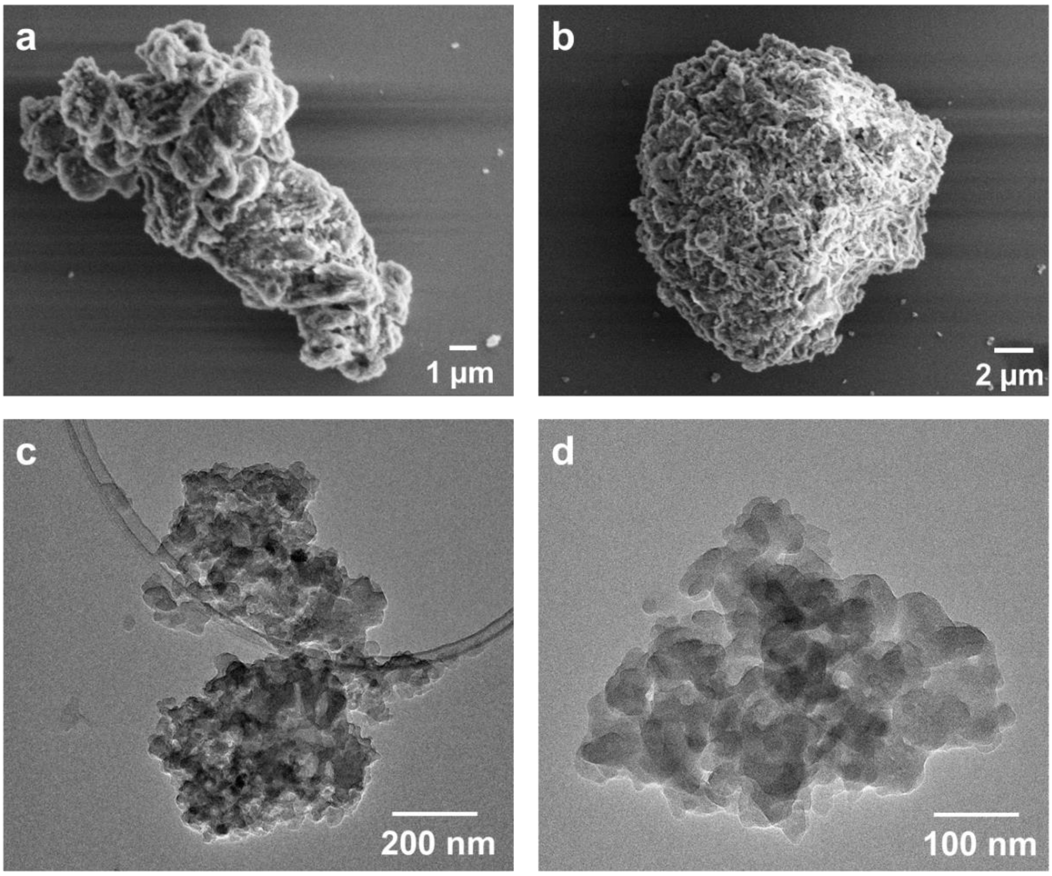

Figure S2. Scanning electron micrographs (a-b) and transmission electron micrographs of **ACA-COF** (c-d).

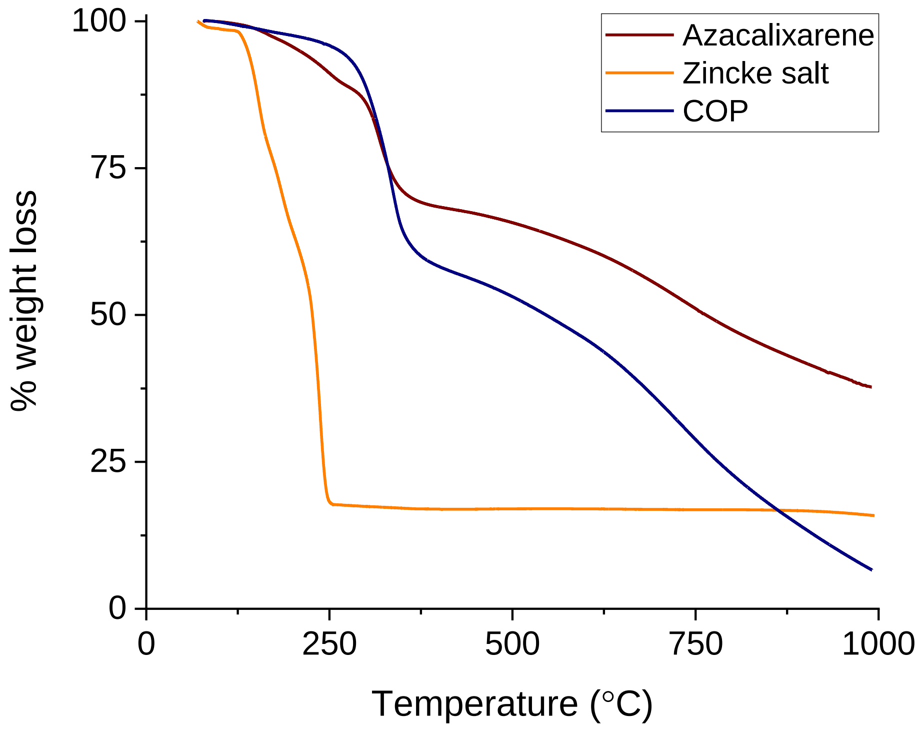

Figure S3. Thermogravimetric analysis of **ACA-COF**, and the corresponding monomers.

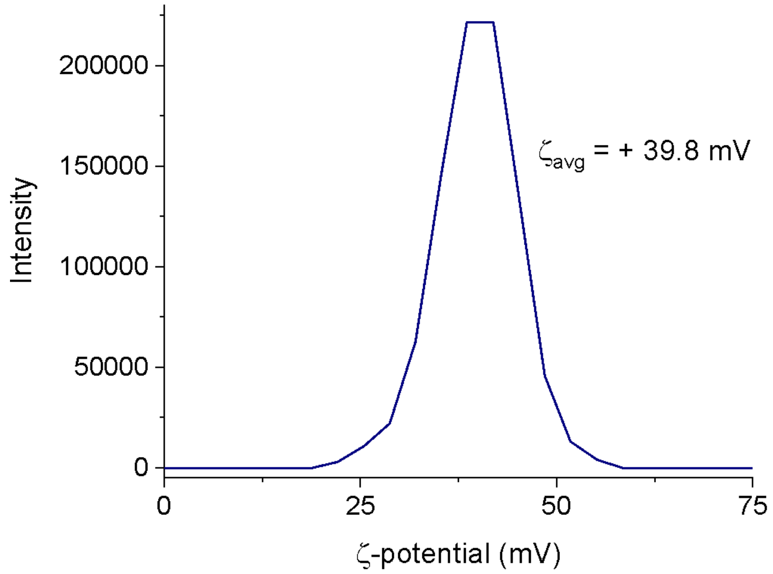

Figure S4. ζ-potential measurement for **ACA-COF** shows an average positive potential of +39.8 mV.

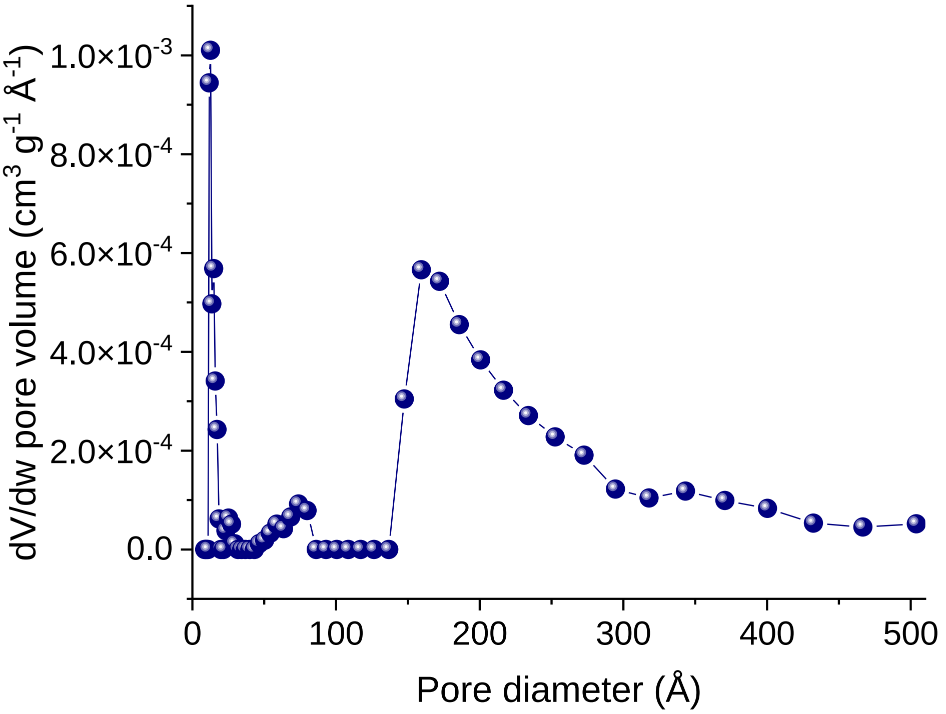

Figure S5. Pore size distribution of **ACA-COF**.

Structural model details

Structural analysis was completed thought computer simulation and powder X-ray diffraction analysis. Crystal structure models were created with the use of Biovia Materials Studio Software. Based on the spectroscopic evidences for bond formation through the Zincke reaction, models based on the generation of extended networks were built up, where the azacalixare units are linked forming 4-connected layers. The models were geometrically optimized following energy minimization procedures, with the use of the Forcite and DFTB+ modules of materials studio. The lattice parameters were initially optimized during the energy minimization procedure, and later refined by completing a full pattern profile (Pawley) refinement with the Reflex module of Materials Studio. The final model consists of an orthorhombic unit cell, space group *Pmmn*, and lattice parameters a = 23.30 Å, b = 12.46, c = 9.85 Å. The obtained atomic coordinates (in cif compatible format) are:

data_AzacalixCOF_Pmmn\Solution_OK

_audit_creation_date 2022-01-20

_audit_creation_method 'Materials Studio'

_symmetry_space_group_name_H-M 'PMMN'

_symmetry_Int_Tables_number 59

_symmetry_cell_setting orthorhombic

```
loop_
_symmetry_equiv_pos_as_xyz
 x,y,z
 -x,-y,z
 -x+1/2,y+1/2,-z
 x+1/2,-y+1/2,-z
 -x+1/2,-y+1/2,-z
 x+1/2,y+1/2,-z
 x,-y,z
 -x,y,z
_cell_length_a              23.2950
_cell_length_b              12.4627
_cell_length_c              9.8510
_cell_angle_alpha           90.0000
_cell_angle_beta            90.0000
_cell_angle_gamma           90.0000
loop_
_atom_site_label
_atom_site_type_symbol
_atom_site_fract_x
_atom_site_fract_y
_atom_site_fract_z
_atom_site_U_iso_or_equiv
_atom_site_adp_type
_atom_site_occupancy
C1   C   0.12614  0.40043  0.00718  0.05000 Uiso  1.00
C2   C   0.17540  0.40057 -0.07866  0.05000 Uiso  1.00
C3   C   0.05244  0.29296  0.14463  0.05000 Uiso  1.00
C4   C   0.05300  0.26328  0.28294  0.05000 Uiso  1.00
N5   N   0.89395  0.69693  0.07239  0.05000 Uiso  1.00
```

N6 N 0.89232 0.25856 0.35439 0.05000 Uiso 1.00

C7 C 0.85641 0.34523 0.34852 0.05000 Uiso 1.00

C8 C 0.80094 0.34265 0.40268 0.05000 Uiso 1.00

C9 C 0.78049 0.25150 0.47117 0.05000 Uiso 1.00

C10 C 0.81980 0.16426 0.48247 0.05000 Uiso 1.00

C11 C 0.87504 0.16907 0.42325 0.05000 Uiso 1.00

N12 N 0.79430 0.69630 -0.12083 0.05000 Uiso 1.00

O13 O 0.82338 0.78509 -0.13627 0.05000 Uiso 1.00

O14 O 0.74159 0.69631 -0.16571 0.05000 Uiso 1.00

H15 H 0.86356 0.75538 0.09773 0.05000 Uiso 1.00

H16 H 0.87004 0.41607 0.29635 0.05000 Uiso 1.00

H17 H 0.77459 0.41209 0.38719 0.05000 Uiso 1.00

H18 H 0.80958 0.09247 0.53759 0.05000 Uiso 1.00

H19 H 0.90377 0.10089 0.43335 0.05000 Uiso 1.00

C20 C 0.10098 0.50000 0.04263 0.05000 Uiso 1.00

C21 C 0.19668 0.50000 -0.12470 0.05000 Uiso 1.00

H22 H 0.06452 0.50000 0.11043 0.05000 Uiso 1.00

H23 H 0.23244 0.50000 -0.19491 0.05000 Uiso 1.00

Cl24 Cl 0.62868 0.50000 -0.12886 0.05000 Uiso 1.00

C25 C 0.00000 0.69288 0.07674 0.05000 Uiso 1.00

C26 C 0.00000 0.24866 0.35132 0.05000 Uiso 1.00

H27 H 0.00000 0.67324 -0.03012 0.05000 Uiso 1.00

H28 H 0.00000 0.23158 0.45877 0.05000 Uiso 1.00

Cl29 Cl 0.00000 0.50000 -0.50118 0.05000 Uiso 1.00

Cl30 Cl 0.00000 0.00000 -0.37552 0.05000 Uiso 1.00

loop_

_geom_bond_atom_site_label_1

_geom_bond_atom_site_label_2

_geom_bond_distance

_geom_bond_site_symmetry_2

_ccdc_geom_bond_type

C1 C2 1.425 . A

C1 C20 1.416 . A

C1 N5 1.450 2_665 S

C2 C21 1.410 . A

C2 N12 1.459 2_665 S

C3 C4 1.412 . A

C3 N5 1.443 2_665 S

C3 C25 1.404 2_565 A

C4 C26 1.418 . A

C4 N6 1.456 8_655 S

N5 H15 1.046 . S

N5 C1 1.450 2_665 S

N5 C3 1.443 2_665 S

N6 C7 1.367 . A

N6 C11 1.366 . A

N6 C4 1.456 8_655 S

C7 C8 1.398 . A

C7 H16 1.070 . S

C8 C9 1.404 . A

C8 H17 1.072 . S

C9 C10 1.426 . A

C9 C9 1.530 5_656 S

C10 C11 1.414 . A

C10 H18 1.073 . S

C11 H19 1.086 . S

N12 O13 1.306 . A

N12 O14 1.305 . A

N12 C2 1.459 2_665 S

C20 C1 1.416 7_565 A

C20   H22   1.080 .   S

C21   C2   1.410 7_565 A

C21   H23   1.083 .   S

C25   H27   1.081 .   S

C25   C3   1.404 2_565 A

C25   C3   1.404 7_565 A

C26   C4   1.418 8   A

C26   H28   1.080 .   S

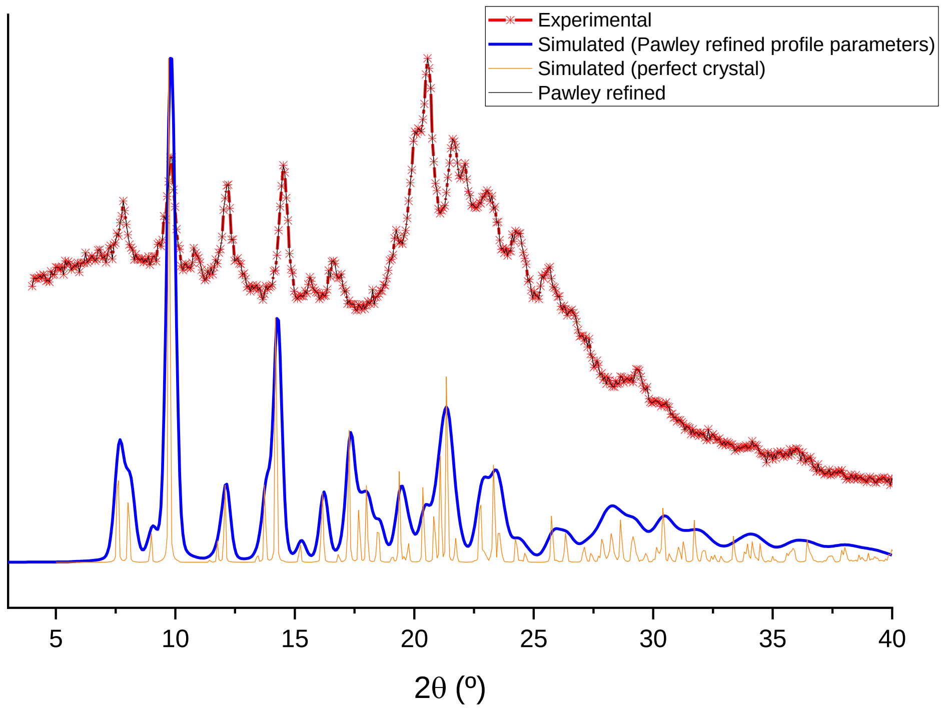

Figure S6. Plot of experimental powder pattern (red), Pawley refined profile (black), pattern calculated for the crystal model including the profile function parameters obtained from Pawley refinement (blue), and pattern calculated for the crystal model, a “perfect crystal” (orange).

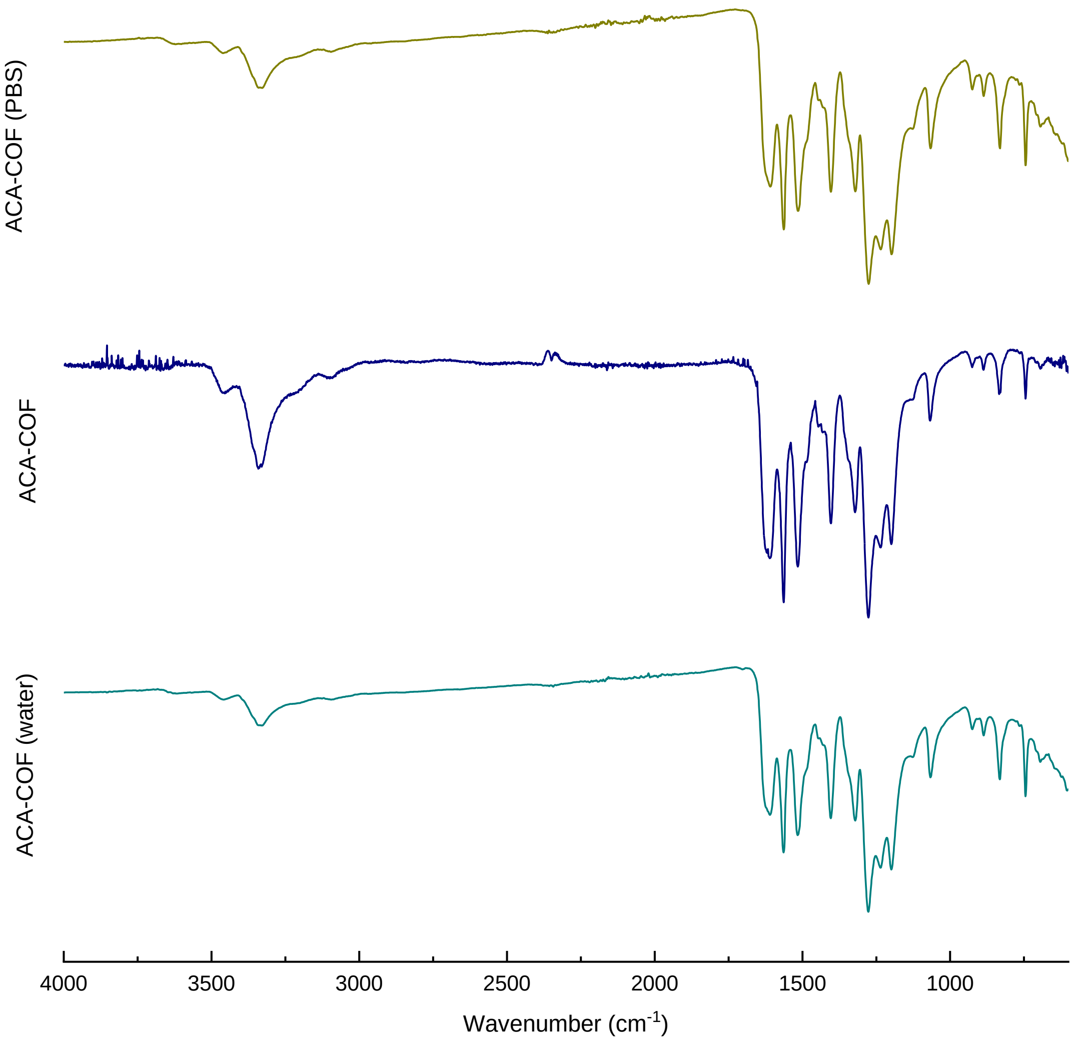

Figure S7. FT-IR spectra of pristine **ACA-COF** (middle), and **ACA-COF** incubated in water (bottom), or PBS (top).

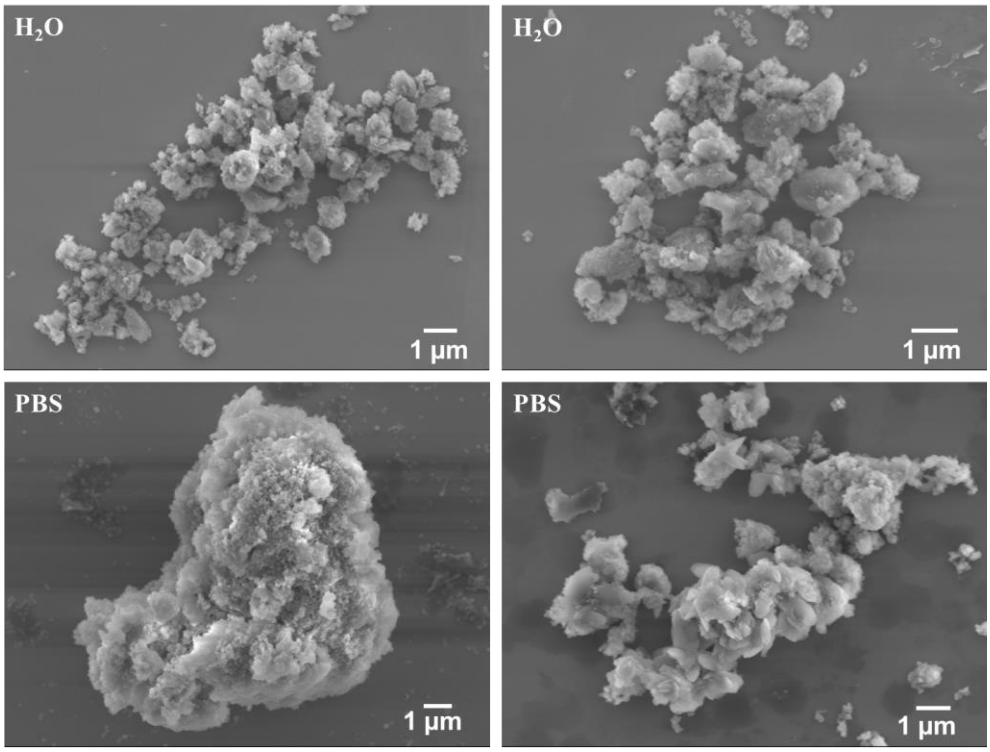

Figure S9. SEM micrographs of **ACA-COF** following a 48-hour incubation in water or PBS at room temperature.

Uric acid and creatinine adsorption experiments

Adsorbate solutions of desired concentrations were obtained by dissolving appropriate amount of either uric acid or creatinine in distilled water. Quantification of uric acid and creatinine was performed on an Agilent 1290 Infinity UHPLC coupled with a Bruker EVOQ triple quadrupole mass spectrometer. Agilent C-18 column (1.8 μm particles) was used with an inner diameter of 2.1 mm and length of 15 mm. Mobile phases consisted of 0.2% formic acid for solvent A and 0.2% formic acid in acetonitrile for solvent B. A sample volume of 10 μL for both calibration standard and unknown was injected onto the column. The samples were eluted from the column in isocratic mode for 3 min with solvent A. A 2 min wash at 80% B was used to keep the column sensitivity high and prevent carry-over, and 2 min equilibration at 0% B was used to regenerate the column for next run. The column was attached to an Agilent UHPLC with a flow rate maintained at 400 μL min^{-1}.

Selected/Multiple reaction monitoring (S/MRM) analyses were carried out on an EVOQ ESI-triple quadrupole mass spectrometer (Bruker) operated in positive ion mode for creatinine and negative ion mode for uric acid. A calibration standard was used for creation of the transitions. Collision energy (CE) was optimized for each transition tested. For uric acid, the final method for S/MRM included the following transitions and specifications: 167.10/96.20 (CE 16 V) and 167.10/124.10 (CE 14 V) where the precursor 167.10 corresponds to uric acid. For creatinine, the final method for S/MRM included the following transitions and specifications: 114.12/44.70 (CE 17 V) and 114.12/86.30 (CE 10 V) where the precursor 114.12 corresponds to creatinine. The rest of the settings for the EVOQ triple quad mass spectrometer were as follows: spray voltage 4500 V, cone temperature 350 °C, cone gas 45 units, heated probe temperature 350 °C, probe gas 25 units, exhaust gas on, and nebulizer gas 25 units. Residual uric acid and creatinine concentrations were determined by means of the linear least square regression model after external calibration with calibration standards (n=5). Calibration standards and samples were run in duplicates, with two blanks run before and after each sample run. The R^2 of the calibration curve was observed to be >0.99.

Adsorption kinetics

The adsorbent (8 mg) was added into a uric acid solution (8 mL, 1.0 mg L^{-1}). The obtained mixture was sonicated briefly before being stirred at room temperature at 400 rpm. At different time intervals, 1 mL was extracted from the solution and passed through a 0.45 µm membrane filter. The remaining amount of the pollutant was determined by HPLC-MS as described above. For experiments involving creatinine, a 2.5 mg L^{-1} solution was used while all other parameters were the same. Syringe filters were found to remove creatinine, so centrifugation was used instead as a means of separating the materials from solutions.

The amount of pollutant at time t, q_t, was calculated by:

$$q_t = \frac{(C_i - C_t) \cdot V \cdot M_w}{m},$$

where q_t (mg g^{-1}) is the quantity of pollutant adsorbed at time t (min), C_i and C_t are the initial and pollutant concentration at time t, M_w is the molecular weight of the pollutant and m (g) is the mass of polymer used for adsorption.

The obtained data was fitted to a pseudo-second-order kinetic model:

$$\frac{t}{q_t} = \frac{1}{k_{obs} q_e^2} + \frac{t}{q_e},$$

where q_t and q_e are the adsorbate uptake per g of adsorbent at time *t* and equilibrium, respectively, (mg g^{-1}) and k_{obs} is the pseudo-second-order rate constant (mg g^{-1} min^{-1}).

Adsorption isotherms

The polymer (1.0 mg) was added into 1.0 mL solution of either uric acid or creatinine of different initial concentrations (0.5, 1.0, 1.5, 2.0, 3.0, 5.0, 7.5, 10.0 mg L^{-1}). The suspension obtained after sonication was stirred overnight until it reached equilibrium. The solutions were filtered through a 0.45 µm membrane filter (uric acid) or centrifuged (creatinine) and the new concentration of paraquat was determined using HPLC-MS.

The amount of pollutant adsorbed at equilibrium q_e was determined by:

$$q_e = \frac{(C_i - C_f) \cdot V \cdot M_w}{m}$$

where C_i and C_f are the initial and final pollutant concentrations (mM), respectively, V is the volume of the solution (L), M_w is the molecular weight of the pollutant, and m is the mass of polymer (g).

The data was fitted to Langmuir isotherm model using the following equation:

$$q_e = \frac{Q_{max} \cdot b \cdot C_e}{1 + b \cdot C_e},$$

where q_e (mg g^{-1}) is the amount of pollutant adsorbed at equilibrium, C_e (mg L^{-1}) is the equilibrium solute concentration remaining in solution when q_e is achieved, Q_{max} is the maximum adsorption capacity corresponding to complete monolayer coverage, and b is a constant (L mg^{-1}).

Table S1. Reported adsorbents for uric acid and their associated rate constants and Q_{max} values.

Material	Rate constant k (g mg^{-1} min^{-1}).)	Q_{max} (mg g^{-1})	Conditions for isotherms	Reference
2-hydroxyethyl methacrylate-ethylene glycol dimethacrylate polymeric granules	n/a	37.0	Sorbent: 50 mg in 10 mL	[3]
ZnO nanoparticles	n/a	345.8	7–35 mg L^{-1}, sorbent 7 – 22 mg	[4]
Chitosan	n/a	17±2	10 mg dL^{-1}, sorbent 0.02 g/10 mL	[5]
Chitosan dialdehyde cellulose	n/a	22±2	10 mg dL^{-1}, sorbent 0.02 g/10 mL	[5]
Fe-chitosan	n/a	10±1	10 mg dL^{-1}, sorbent 0.02 g/10 mL	[5]
Dialdeyde cellulose	n/a	7±1	10 mg dL^{-1}, sorbent 0.02 g/10 mL	[5]
**ACA-COF**	1333.03	5.26	0.5 – 10 mg L^{-1}, sorbent 1 mg/mL	This work

Table S2. Reported adsorbents for creatinine and their associated rate constants and Q_{max} values.

Material	Rate constant k (g mg^{-1} min^{-1})	Q_{max} (mg g^{-1})	Conditions for isotherms	Reference
Zr-based MOF@cotton fabric composite	1.2 × 10^{-3}	212.8	0-900 mg L^{-1}, 1.5 g material, volume 1 L	[6]
Polyacrylonitrile (PAN)-zeolite nanofiber membrane	n/a	25.4	625 µmol L^{-1}, 25 mg sorbent, volume 10 mL	[7]
Mixed-matrix membranes	n/a	234	0–0.15 mg/mL	[8]
Copolymer (HEMA/NVP/MBA) microspheres	n/a	25	n/a	[9]
Organic-inorganic hybrid polymer	n/a	32	5 mg sorbent, volume 5 mL	[10]
Carbon nanotubes	n/a	~25	50 mg sorbent, volume 100 mL, 10 – 100 mg L^{-1}	[11]
Zeolite (mordenite)	n/a	44	0 – 9.5 mM	[12]
Activated carbon	45.20	62.5	40 – 160 mg L^{-1}, volume 20 mL, sorbent 50 mg	[13]
Poly(ether sulfone)/activated carbon	n/a	87	0–80 mg dL^{-1}, volume 20 mL, sorbent 60 beads	[14]

Metal organic framework MIL-100(Fe)	$1.7 \times 10^{-2} \pm 1.6 \times 10^{-3}$	190.5	Sorbent 15 mg, volume 25 mL	[15]
Poly(4-vinylpyridine-*co*-divinylbenzene)	n/a	11.95	Volume 7.5 mL, sorbent 25 g	[16]
Poly-β-cyclodextrin	n/a	6.0	Volume 7.5 mL, sorbent 25 mg	[17]
Polymethacrylic acid	n/a	7.86	Volume 20 mL, sorbent 0.8 g	[18]
Activated carbon	6.86×10^{-4}	38.5	Sorbent 0.1 g, volume 20 mL	[19]
Graphene oxide	9.64×10^{-4}	114.3	Sorbent 0.1 g, volume 20 mL	[19]
Zeolites ZSM-5	1.54×10^{-2}	6.22	Sorbent 0.1 g, volume 20 mL	[19]
Molecularly imprinted polymer	n/a	87.44	15 mg L^{-1}, volume 3 mL, sorbent 1.5 mg	[20]
Dialdehyde nano-fibrillated cellulose	1.608×10^{-3}	16.2	25 – 200 mg L^{-1}, sorbent 0.1 g, volume 25 mL	[21]
Cellulose dinitrobenzoate	9.7×10^{-3}	3.88	10 – 300 mg L^{-1}	[22]
**ACA-COF**	2191.03	1.60	0.5 – 10 mg L^{-1}, sorbent 1 mg/mL	This work

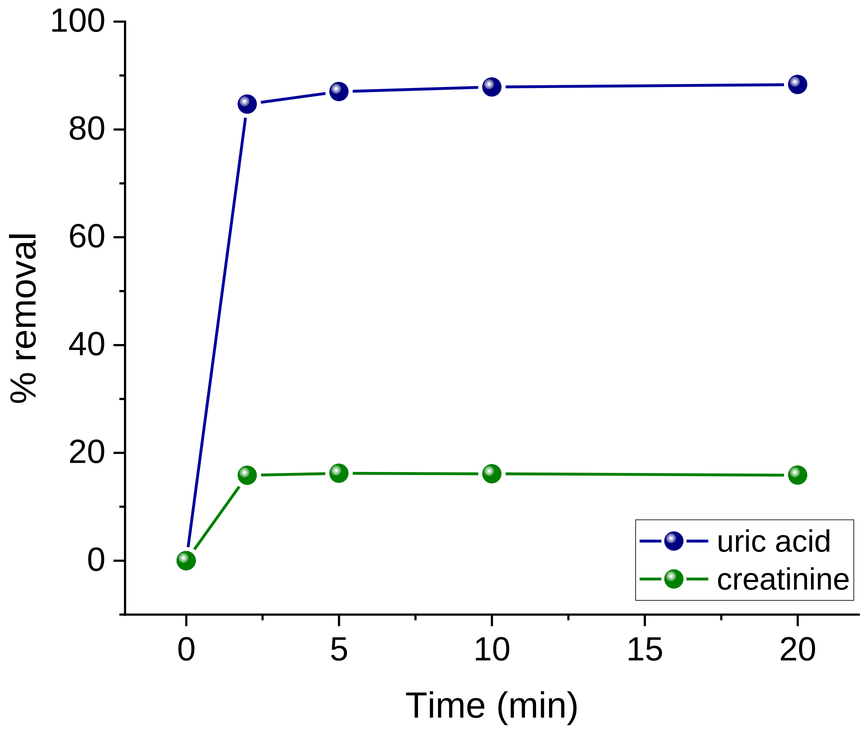

Figure S10. The removal of uric acid and creatinine by **ACA-COF** in presence of 150 mM NaCl.

Molecular Dynamics (MD) simulation setup

The **ACA-COF** polymer was modeled by extending the unit cell of the monomer by 2x2x2 which resulted in a simulation box of 50 Å x 25 Å x 60 Å. A simulation box is created such that at the two dimensions (x, y) the COF is extended to infinity by using periodic boundary conditions. 24 Uric acid (UA) or 24 creatinine (CR) molecules were placed about 35 Å away from the COF surface in random positions to avoid the initial bias in possible binding modes. As a next step, the simulation box was solvated with 1776 water molecules, 24 Cl^- ions for the UA, and 1785 water molecules and 48 Cl^- ions for the creatinine for the neutral systems. For the saline conditions, we used 1762 water, 7 Na^+ ions, and 31 Cl^- ions for the UA system and 1792 water, 7 Na^+ ions, and 55 Cl^- ions for the CR to mimic the 150 mM NaCl salt condition. The Dreiding force field[23] was used to represent water and **ACA-COF**. This forcefield combination has been successfully used in similar studies.[2,24,25] The partial charges for **ACA-COF** were derived by Gasteiger method while the charges of the biological molecules were obtained using QM calculations by fitting the electrostatic potential with ESP module in NWChem.[26] We employed Hartree Fock theory with 6-31G* basis set, keeping all other settings and convergence criteria as default. For ions, we used Joung and Cheatham parameters.[27]

The Gromacs 2018.5 software was employed to perform MD simulations.[28] The initial solvated structure was minimized for 5000 steps and this is followed by 10000 steps of equilibration. In the equilibration process, a position restraint with a stiffness constant of 1000 kJ/mol/A^2 was applied to non-hydrogen atoms of **ACA-COF** and the biological molecule. In the production runs, the position restraints were removed for UA or CR allowing them to freely move around. The equations of motion were solved using the leapfrog integrator[29] with a timestep of 2 fs in a constant temperature and volume (NVT) ensemble. The temperature was maintained at 300 K with the Nose-Hoover thermostat. The Particle Mesh Ewald summation method was used to calculate the electrostatic interactions.[30] 1 nm cut-off distance was maintained for both electrostatic and Van der Walls interactions.

Data analysis

The first 300 ns of the simulation data were discarded to account for the equilibration and the remaining 1.7-μs were used for data analysis. A custom-written python program using MDTraj[31] was used to calculate average percentage removal based on how many biomolecules bind to the **ACA-COF** layers. All solute molecules within a 6 Å distance from the polymer are considered to be bound. Gromacs software tools were used to compute radial distribution functions.

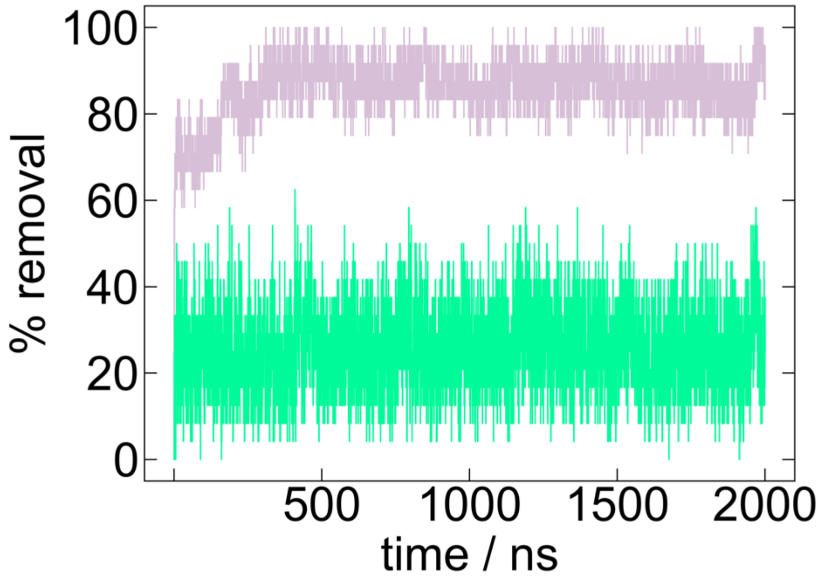

Figure S11. Uptake capacity monitored by the time evolution of percent removal of biomolecules from bulk in the presence of 150 mM NaCl; light purple lines for uric acid and light green for creatinine.

References

[1] Z. Chen, M. Giorgi, D. Jacquemin, M. Elhabiri, O. Siri, *Angew. Chemie Int. Ed.* **2013**, *52*, 6250–6254.

[2] T. Skorjanc, D. Shetty, S. K. Sharma, J. Raya, H. Traboulsi, D. S. Han, J. Lalla, R. Newlon, R. Jagannathan, S. Kirmizialtin, J.-C. Olsen, A. Trabolsi, *Chem. – A Eur. J.* **2018**, *24*, 8648–8655.

[3] A. P. Leshchinskaya, N. M. Ezhova, O. A. Pisarev, *React. Funct. Polym.* **2016**, *102*, 101–109.

[4] F. Marahel, M. Ghaedi, A. Ansari, *Synth. React. Inorganic, Met. Nano-Metal Chem.* **2015**, *45*, 1387–1395.

[5] H. Yoshimoto, S.-B. Jing, T. Yamaguchi, *Bull. Chem. Soc. Jpn* **1995**, 486–489.

[6] R. M. Abdelhameed, M. Rehan, H. E. Emam, *Carbohydr. Polym.* **2018**, *195*, 460–467.

[7] L. Lu, C. Samarasekera, J. T. W. Yeow, *J. Appl. Polym. Sci.* **2015**, *132*.

[8] M. S. L. Tijink, M. Wester, J. Sun, A. Saris, L. A. M. Bolhuis-Versteeg, S. Saiful, J. A. Joles, Z. Borneman, M. Wessling, D. F. Stamatialis, *Acta Biomater.* **2012**, *8*, 2279–2287.

[9] B. Gao, Y. Yang, J. Wang, Y. Zhang, *J. Biochem. Mol. Toxicol.* **2008**, *22*, 166–174.

[10] Y.-S. Chang, T.-H. Ko, T.-J. Hsu, M.-J. Syu, *Anal. Chem.* **2009**, *81*, 2098–2105.

[11] C. Ye, Q.-M. Gong, F.-P. Lu, J. Liang, *Sep. Purif. Technol.* **2007**, *58*, 2–6.

[12] D. Bergé-Lefranc, H. Pizzala, R. Denoyel, V. Hornebecq, J.-L. Bergé-Lefranc, R. Guieu, P. Brunet, H. Ghobarkar, O. Schäf, *Microporous Mesoporous Mater.* **2009**, *119*, 186–192.

[13] Y. Cao, Y. Gu, K. Wang, X. Wang, Z. Gu, T. Ambrico, M. A. Castro, J. Lee, W. Gibbons, J. A. Rice, *J. Taiwan Inst. Chem. Eng.* **2016**, *66*, 347–356.

[14] X. Deng, T. Wang, F. Zhao, L. Li, C. Zhao, *J. Appl. Polym. Sci.* **2007**, *103*, 1085–1092.

[15] C.-X. Yang, C. Liu, Y.-M. Cao, X.-P. Yan, *RSC Adv.* **2014**, *4*, 40824–40827.

[16] H.-A. Tsai, M.-J. Syu, *Anal. Chim. Acta* **2005**, *539*, 107–116.

[17] H.-A. Tsai, M.-J. Syu, *Biomaterials* **2005**, *26*, 2759–2766.

[18] B. Gao, Y. Li, Z. Zhang, *J. Chromatogr. B* **2010**, *878*, 2077–2086.

[19] Y.-C. Cheng, C.-C. Fu, Y.-S. Hsiao, C.-C. Chien, R.-S. Juang, *J. Mol. Liq.* **2018**, *252*, 203–210.

[20] Q. Y. Ang, M. H. Zolkeflay, S. C. Low, *Appl. Surf. Sci.* **2016**, *369*, 326–333.

[21] D. Cui, Z. Liu, Y. Yang, R. Huang, X. Cheng, P. Fatehi, B. Sun, *Biotechnol. Prog.* **2016**, *32*, 208–214.

[22] Q. Dai, J. Ren, W. Kong, F. Peng, L. Meng, *BioResources* **2015**, *10*, 3666–3681.

[23] S. L. Mayo, B. D. Olafson, W. A. Goddard, *J. Phys. Chem.* **1990**, *94*, 8897–8909.

[24] D. Shetty, T. Skorjanc, J. Raya, S. K. Sharma, I. Jahovic, K. Polychronopoulou, Z. Asfari, D. S. Han, S. Dewage, J.-C. Olsen, R. Jagannathan, S. Kirmizialtin, A. Trabolsi, *ACS Appl. Mater. Interfaces* **2018**, *10*, 17359–17365.

[25] B. Garai, D. Shetty, T. Skorjanc, F. Gándara, N. Naleem, S. Varghese, S. K. Sharma, M. Baias, R. Jagannathan, M. A. Olson, S. Kirmizialtin, A. Trabolsi, *J. Am. Chem. Soc.* **2021**, *143*, 3407–3415.

[26] M. Valiev, E. J. Bylaska, N. Govind, K. Kowalski, T. P. Straatsma, H. J. J. Van Dam, D. Wang, J. Nieplocha, E. Apra, T. L. Windus, *Comput. Phys. Commun.* **2010**, *181*, 1477–1489.

[27] I. S. Joung, T. E. Cheatham III, *J. Phys. Chem. B* **2008**, *112*, 9020–9041.

[28] M. J. Abraham, T. Murtola, R. Schulz, S. Páll, J. C. Smith, B. Hess, E. Lindahl, *SoftwareX* **2015**, *1*, 19–25.

[29] W. F. Van Gunsteren, H. J. C. Berendsen, *Mol. Simul.* **1988**, *1*, 173–185.

[30] T. Darden, D. York, L. Pedersen, *J. Chem. Phys.* **1993**, *98*, 10089–10092.

[31] R. T. McGibbon, K. A. Beauchamp, M. P. Harrigan, C. Klein, J. M. Swails, C. X. Hernández, C. R. Schwantes, L.-P. Wang, T. J. Lane, V. S. Pande, *Biophys. J.* **2015**, *109*, 1528–1532.
